# Dissociable mechanisms of speed-accuracy tradeoff during visual perceptual learning are revealed by a hierarchical drift-diffusion model

**DOI:** 10.3389/fnins.2014.00069

**Published:** 2014-04-09

**Authors:** Jiaxiang Zhang, James B. Rowe

**Affiliations:** ^1^Cognition and Brain Sciences Unit, Medical Research CouncilCambridge, UK; ^2^Department of Clinical Neurosciences, University of CambridgeCambridge, UK; ^3^Behavioural and Clinical Neuroscience Institute, University of CambridgeCambridge, UK

**Keywords:** speed-accuracy tradeoff, perceptual learning, drift-diffusion model, Bayesian parameter estimation, motion discrimination

## Abstract

Two phenomena are commonly observed in decision-making. First, there is a speed-accuracy tradeoff (SAT) such that decisions are slower and more accurate when instructions emphasize accuracy over speed, and *vice versa*. Second, decision performance improves with practice, as a task is learnt. The SAT and learning effects have been explained under a well-established evidence-accumulation framework for decision-making, which suggests that evidence supporting each choice is accumulated over time, and a decision is committed to when the accumulated evidence reaches a decision boundary. This framework suggests that changing the decision boundary creates the tradeoff between decision speed and accuracy, while increasing the rate of accumulation leads to more accurate and faster decisions after learning. However, recent studies challenged the view that SAT and learning are associated with changes in distinct, single decision parameters. Further, the influence of speed-accuracy instructions over the course of learning remains largely unknown. Here, we used a hierarchical drift-diffusion model to examine the SAT during learning of a coherent motion discrimination task across multiple training sessions, and a transfer test session. The influence of speed-accuracy instructions was robust over training and generalized across untrained stimulus features. Emphasizing decision accuracy rather than speed was associated with increased boundary separation, drift rate and non-decision time at the beginning of training. However, after training, an emphasis on decision accuracy was only associated with increased boundary separation. In addition, faster and more accurate decisions after learning were due to a gradual decrease in boundary separation and an increase in drift rate. The results suggest that speed-accuracy instructions and learning differentially shape decision-making processes at different time scales.

## Introduction

When making choices under time and resources constraints, more accurate decisions are often achievable at a cost of longer time, while faster responses are more error-prone. This phenomenon of *speed-accuracy tradeoff* (SAT) is ubiquitous across species and tasks (Schouten and Bekker, 1967; Wickelgren, 1977; Chittka et al., 2009), from collective foraging behavior in insects (Chittka et al., 2003; Franks et al., 2003; Marshall et al., 2006) to simple perceptual decisions in mammals (Uchida and Mainen, 2003; Heitz and Schall, 2012), and to complex strategic judgments in human (Beersma et al., 2003).

Most studies on the SAT compare behavioral performance under instructions of speed or accuracy emphasis. Humans can effectively trade accuracy for speed when instructed to respond as fast as possible, or *vice versa* when instructed to respond accurately. A change between speed and accuracy instructions can rapidly switch one's behavior between short blocks of trials (Ratcliff and Rouder, 1998; Mulder et al., 2013) or even between two single trials (Forstmann et al., 2008; Ivanoff et al., 2008), suggesting that such instruction-induced SAT is embodied in the decision-making process. This is consistent with recent findings that the SAT in sensory-motor tasks is associated with neural activities in areas involved in perceptual decisions and cognitive control, such as (pre-) supplementary motor area, the frontal eye field, the anterior cingulate cortex, the striatum, and the dorsolateral prefrontal cortex (Forstmann et al., 2008; Ivanoff et al., 2008; Van Veen et al., 2008; Wylie et al., 2009; Blumen et al., 2011; Heitz and Schall, 2012).

While decisions can be rapidly adjusted in response to speed-accuracy instructions, they are also largely influenced by training and practice over a much longer time frame. It is well-established that prolonged practice gradually improves task performance, resulting in higher accuracy and faster responses (Logan, 1992; Heathcote et al., 2000). Similar to the SAT, the effect of *perceptual learning* is observed across species (Trobalon et al., 1992; Li et al., 2004) and sensory modalities (Fahle and Poggio, 2002), but there are clear distinctions between the two. For simple visual perceptual decisions, performance improvement through perceptual learning is usually specific for the stimuli similar to those used in training, and do not fully generalize to other stimuli when the tasks are difficult (Ahissar and Hochstein, 1997; Green and Bavelier, 2003). Practice on more complex tasks, however, may improve performance in other tasks (Green and Bavelier, 2003). Unlike the SAT, the perceptual learning process can be automatic, without conscious insights of the task. For example, motion discrimination improves as participants were exposed to subliminal motion stimuli when performing an motion-irrelevant task (Watanabe et al., 2001). The specificity, generalizability, and implicit nature of perceptual learning indicate changes in early sensory processing as well as top–down influences during the learning process (Gilbert et al., 2001; Furmanski et al., 2004; Yang and Maunsell, 2004; Fahle, 2005; Bao et al., 2010; Zhang and Kourtzi, 2010; Zhang et al., 2010).

The cognitive processes underpin SAT and perceptual learning have previously been investigated by using the drift-diffusion model (DDM) (Stone, 1960; Ratcliff, 1978). The DDM belongs to a large family of decision-making models, namely sequential sampling models (Wald, 1947; Lehmann, 1959; Stone, 1960; Link, 1975; Link and Heath, 1975; Townsend and Ashby, 1983; Luce, 1986; Ratcliff and Smith, 2004; Smith and Ratcliff, 2004; Bogacz et al., 2006). These models assume that information supporting decisions is represented by a stream of noisy observations over time, and conceptualize decision-making as an information accumulation process: momentary evidence is accumulated over time, which reduce the noise in the evidence and hereby facilitate more accurate decisions. The sequential sampling models have been proven successful in providing a close fit to response accuracy and response time (RT) distributions (e.g., Ratcliff and Rouder, 1998), and are consistent with the identification of putative neural accumulators in the cortex from neurophysiological (Kim and Shadlen, 1999; Shadlen and Newsome, 2001; Roitman and Shadlen, 2002; Schall, 2002; Mazurek et al., 2003; Huk and Shadlen, 2005; Hanks et al., 2006; Gold and Shadlen, 2007) and neuroimage studies (Ploran et al., 2007; Heekeren et al., 2008; Ho et al., 2009; Kayser et al., 2010; Zhang et al., 2012).

**Figure 1:**
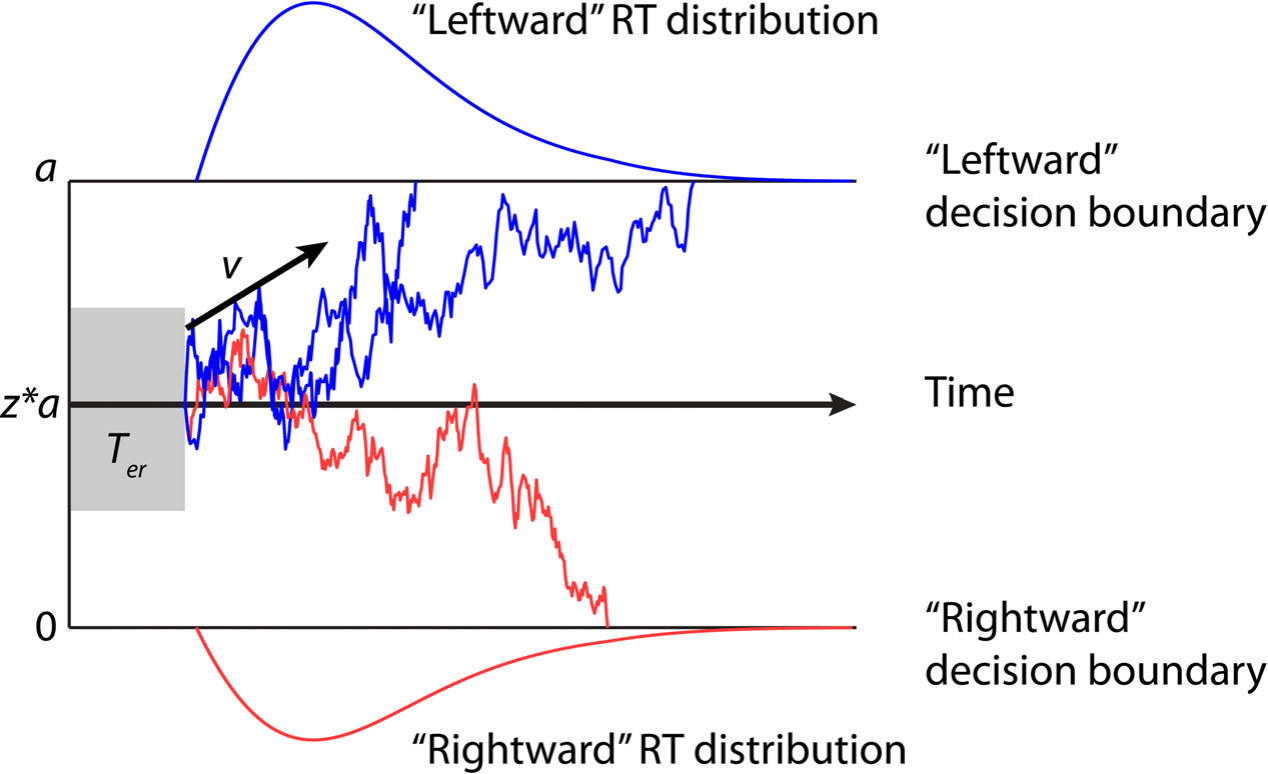
**Examples of trajectories of the drift-diffusion model**. Two decision boundaries (0 and *a*) represent the “leftward” and “rightward” decisions in the motion discrimination task. The drift rate *v* represents mean sensory evidence per unit of time. The magnitude of *v* is determined by the quality of the evidence. A positive *v* (as shown in the figure) indicates that the upper boundary is the correct choice. The diffusion process starts at a starting point between the two boundaries (denoted as a proportion of *a* by *z*) until the accumulated evidence reaches one of two boundaries. If the correct boundary is hit (blue sample paths), the model makes a correct decision. Because of noise, the model may sometime hit the incorrect boundary (red sample path). The predicted response time (RT) is the sum of the duration of the diffusion process and the non-decision time *T*_*er*_.

The DDM is one of the most prominent sequential sampling models for two-choice decisions. It has been applied to a number of perceptual and cognitive tasks, including memory retrieval (Ratcliff, 1978), lexical decisions (Ratcliff et al., 2004; Wagenmakers et al., 2008), visual discrimination (Ratcliff, 2002; Palmer et al., 2005), and categorization (Nosofsky and Palmeri, 1997). The model implies a single accumulator integrating the sample evidence according to a stochastic diffusion process, until the accumulated evidence reaches one of the two decision boundaries, corresponding to the two choice alternatives. As such the model decomposes behavioral data into four parameters mapped on to latent psychological processes (Figure 1): boundary separation *a* for response caution, drift rate *v* for speed of accumulation, starting point *z* for *a priori* response bias, and non-decision time *T*_*er*_ for stimulus encoding and response execution latencies (Ratcliff and McKoon, 2008; Wagenmakers, 2009). Trial-to-trial variability in model parameters can be included to improve the model fits to experimental data (Laming, 1968; Ratcliff, 1978; Ratcliff et al., 1999; Ratcliff and Tuerlinckx, 2002).

Behavioral changes in SAT and perceptual learning can be explained by different parameter changes in the DDM. The SAT can be simply quantified by the separation of the two decision boundaries. When response speed is emphasized, the distance between decision boundaries is decreased. This reduces the amount of accumulated evidence prior to a decision (i.e., faster RT) and increase the change of hitting the wrong decision boundary (i.e., lower accuracy). When accuracy is emphasized, the distance between decision boundaries is increased and the model predicts slower RT and higher accuracy, because more evidence need to be accumulated prior to a decision. It has indeed been shown that emphasizing decision speed or accuracy leads to changes in the boundary separation (Ratcliff and Rouder, 2000). A few recent studies have also applied the DDM to perceptual learning and identified two separate learning mechanisms (Dutilh et al., 2011, 2009; Petrov et al., 2011). First, training and practice are associated with an increase in the drift rate, leading to higher accuracy and faster RT (Dutilh et al., 2009; Wagenmakers, 2009). The drift rate change is consistent with most learning theories that the quality of sensory processing improves during training (Ahissar and Hochstein, 2004). Second, perceptual learning has been shown to decrease the non-decision time, which may be due to an increase in familiarity with the stimuli and task after training (Dutilh et al., 2011, 2009; Petrov et al., 2011).

However, two important issues remain unsolved. First, although previous research proposed that emphasizing speed or accuracy influence only the boundary separation (Ratcliff and Rouder, 1998; Wagenmakers et al., 2008), recent studies showed that speed-accuracy instructions affect two other model parameters: drift rate (Vandekerckhove et al., 2011; Rae et al., in press) and non-decision time (Osman et al., 2000; Rinkenauer et al., 2004; Voss et al., 2004; Mulder et al., 2010, 2013). Therefore, it is necessary to examine whether other model parameters are indeed affected by speed emphasis or accuracy emphasis instructions.

Second, previous studies of the SAT and perceptual learning have been largely independent, partly because of the different time scale on which the two effects operate. However, since speed-accuracy instructions and learning can affect the same decision parameters, it is necessary to study these two different task conditions in a single experiment. Here we test the intriguing hypothesis that the SAT be efficiently manipulated over the course of learning a new task. One might establish a stable tradeoff between speed and accuracy throughout learning, according to the task instructions. Alternatively, the effects of speed-accuracy instructions in a new task may be different from that in the same task after substantial practice.

**Figure 2:**
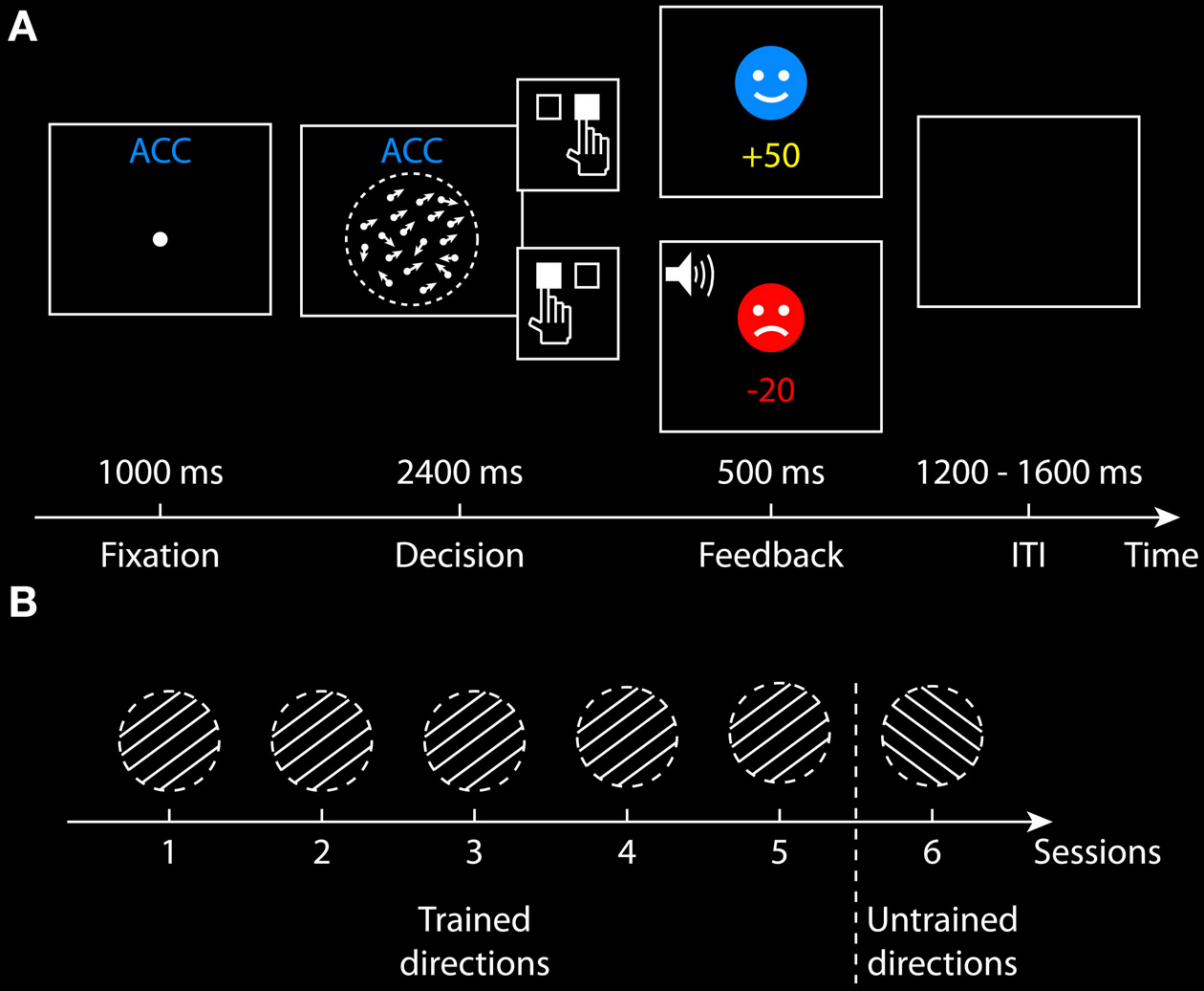
**Behavioral paradigm. (A)** Structure of a single trial in the accuracy condition. A fixation point was presented for 1000 ms. The random dot kinematogram was then presented for a maximum of 2400 ms, during which participants made a binary decision on whether the coherent motion direction is leftward or rightward by pressing one of the two response buttons. For a correct response, a smiley face was presented for 500 and 50 points was credited. For an incorrect response, a sad face was presented and 20 points was lost, together with an auditory feedback. The payoff in the speed condition was slightly different (see section Task and Procedurefor more details). The intertrial interval (ITI) was randomized between 1200 and 1600 ms. **(B)** Training procedure across six sessions. In the first five sessions, half of the participants trained at two directions (30 and 210°), and the other half trained at two different directions (150 and 330°). In the sixth session, all participants performed the task at two new directions that were not presented in their first five sessions (i.e., untrained directions).

The current study examined changes in decision performance and underlying cognitive mechanisms when SAT was manipulated throughout the course of learning. During multiple training sessions, participants learned to perform a coherent motion discrimination task under speed or accuracy emphasis (Figure 2A). Speed-accuracy instructions efficiently modulated participants' behavior between short blocks of trials across all sessions and training gradually improves performance specific to the trained directions. By fitting the DDM using Bayesian parameter estimation approach, we quantified the influence of speed-accuracy instructions and learning on the model parameters. Emphasizing decision accuracy rather than speed was related to increased boundary separation, drift rate and non-decision time at the beginning of training. In contrast, the emphasis on accuracy was only related to increased boundary separation after training. Furthermore, faster and more accurate decisions after learning are mainly due to a decrease in boundary separation and an increase in drift rate. Our results demonstrate that decision-making processes are differentially influenced by speed-accuracy instructions and training at different time scales and different stages of learning.

## Materials and methods

### Participants

Six adults (four females) between the age of 21–35 years (mean age, 25.50 years) participated in the experiment. All participants were right handed with normal hearing and normal or corrected-to-normal vision, and none reported a history of significant neurological or psychiatric illness. None had previous experience with the task. All participants signed a written informed consent before starting the experiment. The study was approved by the Cambridge Psychology Research Ethics Committee.

### Apparatus

The experiment was conducted in a darkened testing room. Each participant's head rested in a chinrest to stabilize the head position and control viewing distance. A computer (Dell Optiplex 745) controlled stimulus delivery and recorded behavioral responses. Visual stimulus was presented on a 21-inch CRT monitor (Dell P1130) with a resolution of 1024 by 768 pixels and a refresh rate of 85 Hz, located 47.50 cm in front of the participants. Participants' responses were collected from a two-button response box. The experiment was written in Matlab 7.8 (The MathWorks, Natick, USA) and used the Psychophysics Toolbox 3 extensions (Brainard, 1997).

### Stimuli

The stimuli were random-dot kinematograms displayed within a central invisible circular aperture (12° diameter) on a black background (100% contrast). Dot density was 16.53 dots per deg^2^ per s and the minimum distance between any two dots in each frame was 0.48°. Each dot was white and subtended a visual angle of 0.12° at the screen center. The motion stimulus was formed by interleaving three uncorrelated sequences of dot positions at a rate of 85 frames/s, which was similar to those described elsewhere (Britten et al., 1993; Shadlen and Newsome, 2001; Roitman and Shadlen, 2002; Pilly and Seitz, 2009). To introduce coherent motion information, in each frame a fixed proportion (10.71%) of the dots was replotted at an appropriate spatial displacement in the direction of motion (10°/s velocity), relative to their positions three frames earlier, and the rest of the dots were replotted at random locations within the aperture. For example, three uncorrelated sets of dots were plotted in the first three frames. A proportion of dots (i.e., the signal dots) in frame 1 moved in frame 4 with spatial displacements, and then a proportion of dots in frame 2 moved in frame 5, and so on. Signal dots that moved outside the aperture were wrapped around from the opposite direction of motion to conserve dot density and avoid attention cues along edges. The coherent dot motion in each trial was in one of four non-cardinal directions (30, 150, 210, and 330°).

### Task and procedure

All participants completed six behavioral sessions conducted on different days. Participants performed a two-alternative forced-choice task in all sessions, deciding whether the coherent motion direction of the random-dot stimulus is leftward (toward 150 or 210°) or rightward (toward 30 or 330°) (Figure 2A). Participants responded by pressing the left button (for leftward decisions) or the right button (for rightward decisions) on the response box with their right index and middle fingers. In the first five sessions, the random-dot stimulus was always presented at two possible directions along a line (e.g., 30 and 210°), which referred to as the trained directions. In the sixth session, the stimulus was only presented at the other two new directions (e.g., 150 and 330°), which referred to as the untrained directions. One-half of the participants were trained at 30 and 210° directions and the other half of the participants were trained at the 150 and 330° directions in their first five sessions (Figure 2B).

Each experiment session comprised 672 trials, which were divided into 12 blocks of 56 trials. Each block had 50% leftwards motion trials and 50% rightwards motion trials at a randomized order. Participants took short breaks between blocks. The speed-accuracy manipulation was introduced at the block level: each session comprised of 6 accuracy blocks and 6 speed blocks. The first block of each session was always an accuracy block, and the order of the accuracy/speed instructions in the rest of the blocks were randomized across sessions and participants. At the beginning of an accuracy block, the text instruction “*Be accurate this time*” was presented on the screen in blue (RGB = 5, 137, 255), indicating that the participants should respond as accurate as possible. At the beginning of a speed block, the text instruction “*Be fast this time*” was presented in red (RGB = 255, 2, 2), indicating that the participants should respond as fast as possible. To ensure participants could easily identify the task instructions during the experiment, a text cue was presented at the top center of the screen throughout each block: “ACC” in blue (RGB = 5, 137, 255) for accuracy blocks, and “SPD” in red (RGB = 255, 2, 2) for speed blocks. Before the first and the 29th trials of each block, four parallel gray lines (RGB = 100, 100, 100, 0.05° thick, 4° apart) were presented within the circular aperture for 2000 ms, indicating the two possible motion directions in the current block (30 and 210°, or 150 and 330°). Before the first session, each participant was familiarized with the task during a short practice run comprising 16 trials for the accuracy condition and 16 trials for the speed condition, during which the proportion of coherently moving dots was set at a high level of 80%.

Each trial began with the presentation of a fixation point (0.12° diameter) at the center of the screen, which was illuminated for 1000 ms, followed by the random-dot stimulus onset. The stimulus was presented for a maximum of 2400 ms, during which the participants were instructed to perform the motion discrimination task under accuracy or speed emphasis. The random-dot stimulus disappeared as soon as a response was made, or the maximum duration was reached. The RT on each trial was measured from the stimulus onset until the participant made a response. Feedback was given 100 ms after the stimulus offset, followed by an intertrial interval randomized between 1200 and 1600 ms (Figure 2A).

To help the participants engage in the task and effectively adjust their decision processes to the speed-accuracy instructions, three types of feedback were given in the forms of texts, auditory beeps (tone with frequency of 600 Hz and duration of 0.15 s), and bonus points (see Petrov et al., 2011; Mulder et al., 2013 for similar multi-session designs using bonus points). If the participant failed to respond within 2200 ms or responded within 100 ms, a red warning message “Too slow!” or “Too fast!” was presented for a prolonged period (1500 ms) together with a beep, and the participant lost 50 points. In the accuracy condition, if the participant made a correct response, a smiley face was presented for 500 ms and 50 bonus points were credited. For an incorrect response, a sad face was presented for 500 ms and a beep where given, and the participant lost 20 points. In the speed condition, when the participants failed to respond within a time limit, a red text “Too slow!” and a beep was given and the participant lost 20 points. No further feedback about the accuracy of the participants' responses was given (i.e., they would also lose 20 points for a correct but overtime response). For each session and each participant, the time limit for the speed condition was defined as the 40% quantile of the RT distribution from the participant's first accuracy block in that specific session (see Mulder et al., 2013 for another way of defining participant-specific time limit). If participant's response was within the time limit, the same type of feedback was given for correct and incorrect responses as in the accuracy condition, but the participant would only lose 10 points for an incorrect response (i.e., fewer penalties for errors when instructing speeded responses). Participants started with zero bonus point at the beginning of each session and the cumulative bonus points were displayed at the bottom of the screen throughout the session.

### Data processing and analysis

To eliminate fast guesses, trials with RT faster than 100 ms were removed from further analysis. Trials without a valid response within 2200 ms after the random-dot stimulus onset were also removed. The discarded trials only accounted for 0.3% of all trials. Decision accuracies (proportion of correct responses) and mean RTs from each session were entered into two separate repeated-measures ANOVAs for group analyses, with task conditions (accuracy and speed instructions) and sessions as factors.

Randomization tests were used to examine the statistical significance at the single-subject level (Edgington, 1995; Coolican, 2009). For example, to test whether a single participant had different RT between the speed and accuracy conditions, we first estimated the mean RT separately from each block in each session of the participant, resulting in RT samples from 36 speed blocks and 36 accuracy blocks. The observed RT difference between the two task conditions was quantified by the sample *t*-value (mean difference between the data from the speed emphasis and accuracy emphasis conditions divided by the standard error of the difference). If the null hypothesis is true, there is no difference between task conditions, and the samples are exchangeable between conditions. We therefore generated a null distribution of the test statistic from 100,000 permutations, with the condition label randomly shuffled in each permutation. The permutation *p*-value was then calculated as the proportion of the randomized samples with the test statistic exceeded the observed test statistic. The same randomization procedure was applied to test the learning effects between sessions (Table 1).

**Table 1:** **Results of single-subject randomization tests**.

**Participant**	**SAT effects**				**Learning effects**				**Learning generalization**			
	**Accuracy**		**RT**		**Accuracy**		**RT**		**Accuracy**		**RT**	
	**(ACC > SPD)**		**(ACC > SPD)**		**(session 1–5)**		**(session 1–5)**		**(session 5–6)**		**(session 5–6)**	
	***t***	***p***	***t***	***p***	***t***	***p***	***t***	***p***	***t***	***p***	***t***	***p***
S01	1.92	0.0600	2.05	0.0398	−2.82	0.0090	3.84	<0.0001	2.23	0.0348	3.14	0.0057
S02	1.85	0.0695	3.46	0.0007	−3.89	0.0012	4.57	<0.0001	3.90	0.0008	2.83	0.0102
S03	3.38	0.0015	9.73	<0.0001	−1.74	0.0963	2.45	0.0237	1.28	0.2121	−0.37	0.7150
S04	4.35	<0.0001	5.06	<0.0001	−3.44	0.0028	2.43	0.0194	2.31	0.0336	−0.53	0.6114
S05	2.40	0.0200	10.89	<0.0001	2.40	0.0258	2.73	0.0132	1.70	0.1093	−0.69	0.5077
S06	3.99	0.0002	4.37	<0.0001	−3.52	0.0018	6.50	<0.0001	2.78	0.0110	−1.20	0.2508

*The SAT effects compared the behavioral performance between accuracy and speed conditions across all sessions. The learning effects compared the performance between session 1 and 5. The learning generalization effects compared the accuracy and RT between session 5 and 6 (i.e., performance at the untrained directions). Differences between conditions were quantified by sample t-values. Each p-value was obtained from 100,000 permutations of data samples (see section Data Processing and Analysis for details)*.

### Hierarchical drift-diffusion model

A full version of the DDM was fitted to each participant's accuracy and RT distribution. The model consists of seven parameters (Ratcliff and McKoon, 2008; Wagenmakers, 2009). (1) Boundary separation *a* (*a* > 0). (2) Mean drift rate *v*. (3) Mean response bias *z* as a proportion of boundary separation (0 < *z* < 1), which gives the starting point of the diffusion process relative to the two boundaries (z*a). Thus, values of *z* > 0.5 indicate an *a priori* bias toward the upper boundary (right button press) and values of *z* < 0.5 indicate a bias toward the lower boundary (left button press). (4) Mean non-decision time *T*_*er*_. (5) Normally distributed trial-by-trial variability in drift rate *s*_*v*_. (6) Uniformly distributed trial-by-trial variability in response bias *s*_*z*_. (7) Uniformly distributed trial-by-trial variability in non-decision time *s*_*t*_. The model predicts a binary choice as whether the upper or the lower boundary is reached, and predicts the observed RT as a sum of the decision time (i.e., the latency for the accumulator reaching one of the boundaries) and the non-decision time.

We used the hierarchical drift-diffusion model toolbox to fit the data (Wiecki et al., 2013). The hierarchical extension of the DDM assumes that the model parameters for individual participants are random samples drawn from group-level distributions, and uses Bayesian statistical methods to simultaneously estimate all parameters at both the group level and the individual-subject level (Vandekerckhove et al., 2011). The Bayesian approach for parameter estimation has two advantages. First, the Bayesian approach is more robust in recovering model parameters when less data is available (Matzke et al., 2013; Wiecki et al., 2013). Second, Bayesian estimation generates joint posterior distributions of all model parameters, given the observed experimental data. The posterior parameter distribution provides not only a point estimate, but also uncertainty of the estimate, and can be straightforwardly applied for Bayesian inference (Gelman et al., 2004). For example, let *P*_Post|Data_(*a*_accuracy_) and *P*_Post|Data_(*a*_speed_) be the marginal posteriors for the boundary separation from the accuracy and speed conditions. To test whether the boundary separation in the accuracy condition is larger than that in the speed condition, we can directly calculate the probability that the difference between the two parameters is larger than zero *P*_Post|Data_(*a*_accuracy_ – *a*_speed_ > 0) from the posterior distributions, and a high probability indicates strong evidence in favor of the testing hypothesis.

Performance differences between speed-accuracy conditions and between sessions suggest changes in one or more model parameters across task conditions and sessions. We therefore examined seven variants of the DDM with different parameter constrains between the two task conditions. The seven models differed on whether the boundary separation *a*, the drift rate *v*, the non-decision time *T*_*er*_, or a combination of the three parameters varied between the accuracy and speed conditions (Figure 4). In all the models, the four key parameters (*a, v, T*_*er*_, and *z*) were allowed to vary between sessions and were estimated at both individual-subject level and group level. The trial-by-trial variability parameters (*s*_*v*_, *s*_*t*_, and *s*_*z*_) were shared between sessions and were estimated only at the group level, because it has been shown that the DDM with variability parameters fixed across multiple sessions provided a better explanation of the data (Liu and Watanabe, 2012). Similar to previous studies, the response bias parameter was set to vary between sessions but was invariant between task conditions (Mulder et al., 2013).

For each model, we generated 15,000 samples from the joint posterior distribution of all model parameters by using Markov chain Monte Carlo methods (Gamerman and Lopes, 2006) and discarded the first 5000 samples as burn-in (see Wiecki et al., 2013 for a more detailed description of the procedure). The convergence of the Markov chains were assessed using Geweke statistic (Gelman and Rubin, 1992). Parameter estimates in all models were converged after 15,000 samples.

## Results

### Speed-accuracy tradeoff and learning effects on behavioral performance

**Figure 3:**
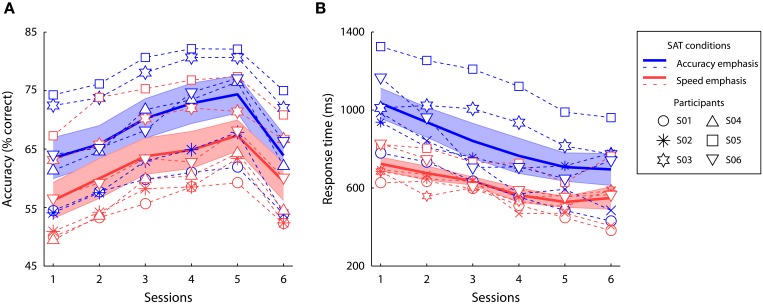
**Behavioral results**. Decision accuracy **(A)** and mean response time **(B)** of the speed emphasis and accuracy emphasis conditions at each training session. The solid lines and the shaded areas indicate the mean performance and the standard errors across participants. Different markers indicate performance of each individual participant across sessions.

Participants' performance under the accuracy and speed conditions was quantified by the mean decision accuracy and mean RT in each session (Figure 3). A two-way repeated-measures ANOVA showed a significant main effect of speed-accuracy instructions [accuracy: *F*_(1, 5)_ = 27.57, *p* < 0.01, partial η^2^ = 0.85; RT: *F*_(1, 5)_ = 17.56, *p* < 0.01, partial η^2^ = 0.78], a significant main effect of session [accuracy: *F*_(5, 25)_ = 67.48, *p* < 0.0001, partial η^2^ = 0.93; RT: *F*_(5, 25)_ = 22.82, *p* < 0.0001, partial η^2^ = 0.82], and a significant interaction between speed-accuracy manipulation and session [accuracy: *F*_(5, 25)_ = 4.78, *p* < 0.01, partial η^2^ = 0.49; RT: *F*_(1, 5)_ = 5.08, *p* < 0.01, partial η^2^ = 0.50]. In each session, the participants had higher accuracy (*p* < 0.05 in all sessions, Wilcoxon signed ranks test) and faster RT (*p* < 0.05 in all sessions, Wilcoxon Signed Ranks Test) in the accuracy condition than in the speed condition. Therefore, throughout training, the participants could effectively trade speed for accuracy (and *vice versa*) as instructed.

During the first five training sessions, behavior performance at the trained directions gradually improved, as shown by a significant linear increase of accuracy [*F*_(1, 5)_ = 102.07, *p* < 0.0001, partial η^2^ = 0.95] and a linear decrease of RT [*F*_(1, 5)_ = 53.37, *p* < 0.001, partial η^2^ = 0.91] over training. To examine whether the behavioral improvement at the trained directions can be generalized to another direction, we compared participants' performance between the 5th session (i.e., the last session at the trained directions) and the 6th session (i.e., untrained directions after training). The learning effect on decision accuracy was specific to individual participants' trained directions, as the accuracy was significantly lower at the untrained directions than the trained directions [*F*_(1, 5)_ = 73.56, *p* < 0.0001, partial η^2^ = 0.94]. Further, the learning effect on decision speed generalized across directions, as the RT at the untrained directions did not significantly differ to the trained directions after training [*F*_(1, 5)_ = 0.03, *p* = 0.87, partial η^2^ = 0.01], but much faster than that in the first session [*F*_(1, 5)_ = 35.94, *p* < 0.01, partial η^2^ = 0.88].

These results indicate strong group effects of speed-accuracy instructions and learning in perceptual decisions. Since the experiment collected substantial amount of data from individual participants, it is effective to further examine whether each individual's performance is consistent with the group effects above (Coolican, 2009; Barnett et al., 2012). We therefore conducted single-subject randomization tests (Bulté and Onghena, 2008, see section Data Processing and Analysis for details), estimating the main effects of task instructions across all sessions, the effect of learning, and generalization between trained and untrained directions for each participant (Table 1). Four participants had significantly higher decision accuracy and slower RT across sessions when instructed to trade speed for accuracy, with a trend effect in the accuracy in two participants (S01 and S02 in Table 1). After training, significant improvements in both accuracy and RT were observed in five out of six participants, except one participant (S03) who had faster RT but no significant accuracy change after training. Four participants had significantly lower accuracies at the untrained directions than the trained directions after training. These analyses suggested that the single-subject data are largely consistent with the group inferences.

### Hierarchical drift-diffusion model for speed-accuracy tradeoff and learning

To examine which model parameters account for the effects of speed-accuracy instructions during learning, we considered seven variants of the hierarchical DDM, varying systematically in constraints on whether three model parameters (*a, v*, and *T*_*er*_) were invariant or varied across the task conditions. We used a Bayesian parameter estimation procedure to draw samples from the joint posterior distributions of all the parameters in the hierarchical DDM (Vandekerckhove et al., 2011; Wiecki et al., 2013). The posterior samples represents parameter estimates and their uncertainties after having observed the data (i.e., response and RT distributions) (Gelman et al., 2004). Model fits were assessed by comparing each model's deviance information criterion (DIC) value (Spiegelhalter et al., 2002), which has a degree of penalty for additional free model parameters.

**Figure 4:**
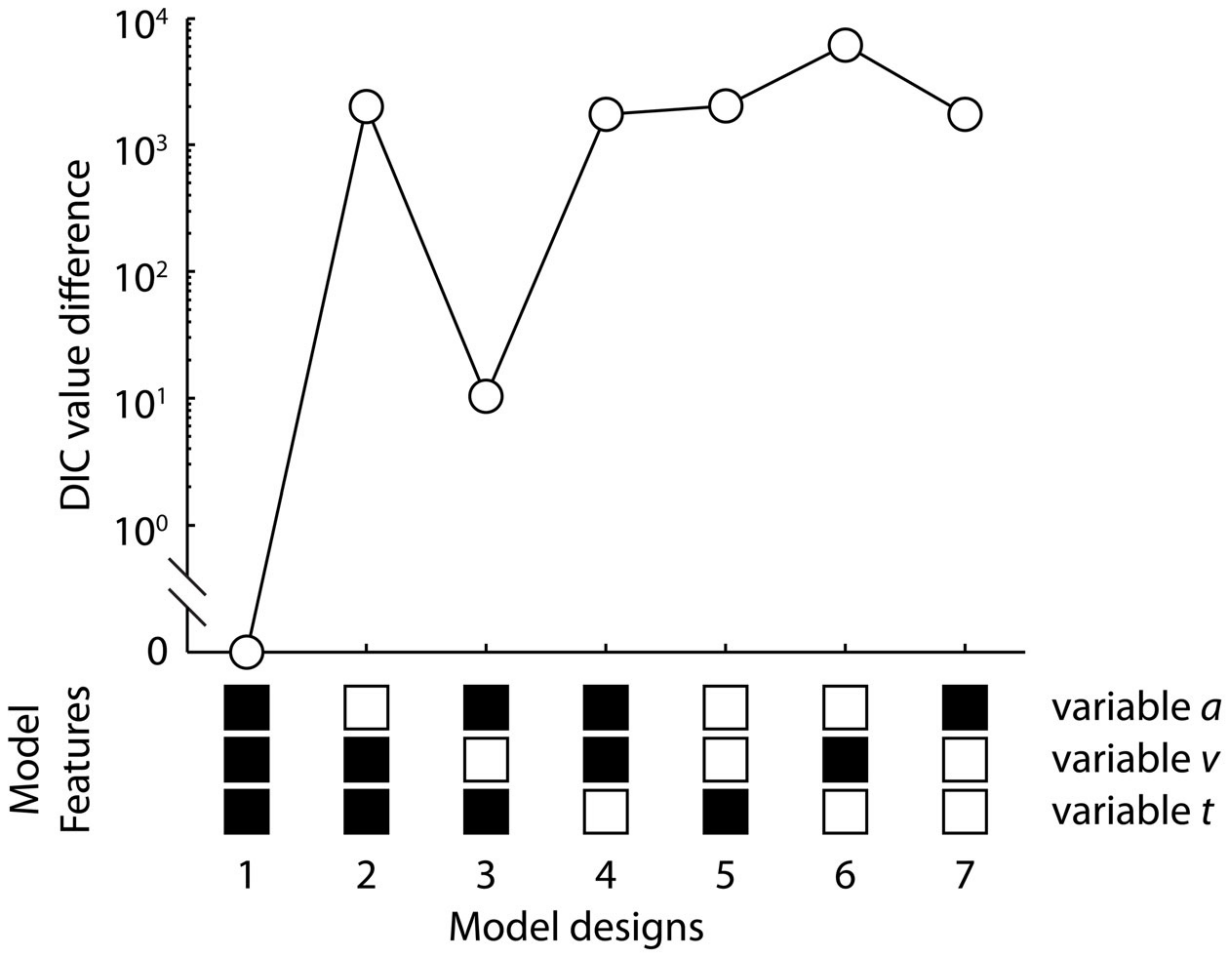
**The deviance information criterion (DIC) value differences between the seven variants of the drift-diffusion model and the best model**. The models differ on whether the boundary separation *a*, mean drift rate *v*, and mean non-decision time *T*_*er*_ can vary between the speed and accuracy conditions. The model structures are shown below the figure. The black square indicates that the corresponding parameter can vary between the speed emphasis and accuracy emphasis conditions, and the white square indicates that the parameter is invariant between the two task conditions. The best model with the minimum DIC value had variable *a, v*, and *T*_*er*_ (model 1, DIC = 9474.03).

The best model (the one with the lowest DIC value) to describe the data across task conditions, sessions and participants allows the boundary separation *a*, mean drift rate *v*, and mean non-decision time *T*_*er*_ all to vary between speed and accuracy conditions (model 1 in Figure 4). The second best model had varied *a* and *T*_*er*_ but invariant *v* between SAT conditions, which had a DIC value 10.37 larger than the best model (model 3 in Figure 4). The model with only varied *v* but invariant *a* and *T*_*er*_ (model 6 in Figure 4) provided the worst fit among the seven models. Thus, changes in the mean drift rate are less likely to significantly account for the observed speed-accuracy effects. In later analysis, we focused on the best model with the minimum DIC value^1^.

**Figure 5:**
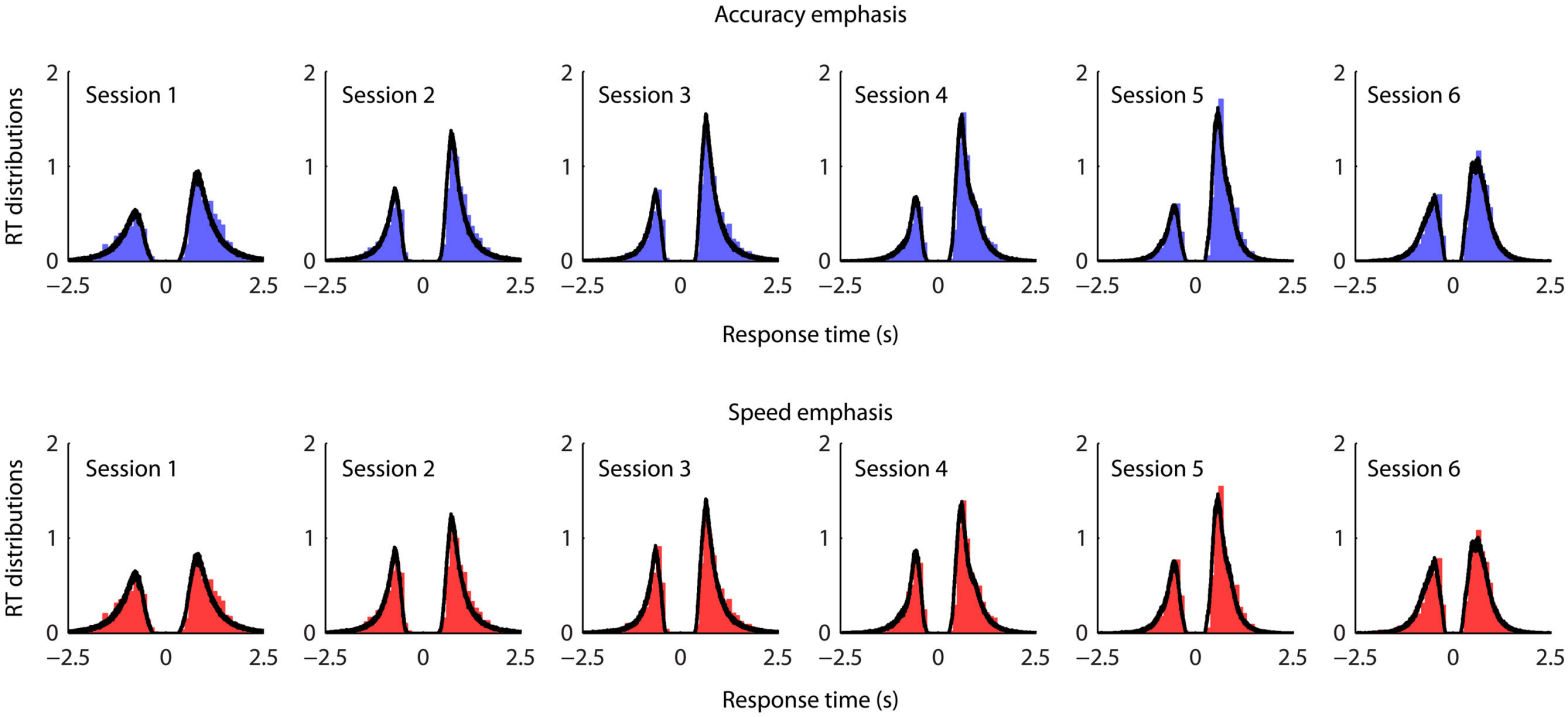
**Posterior predictive data distributions for the task conditions and sessions from the best fit model**. The distributions along the positive *x*-axis indicate correct response times, and the distributions along the negative *x*-axis indicate error response times. Each panel shows the normalized histograms of the observed data (bar plots) and the model prediction (black lines). The area under the curve at positive *x*-axis is therefore corresponding to the observed and predicted proportion correct. To generate model predictions, for each participant and each model parameter, we drew 500 sampled values from that participant's joint posterior distribution of the model parameters, which give 500 posterior parameter sets. Each sampled parameter set was then used to simulate the same amount of model-predicted data as observed in the experiment. The simulated RT distributions of correct and error trials were then averaged across the parameter sets as posterior model predictions. Data from individual participants are pooled together.

To evaluate the overall model fit, we generated posterior model predictions of the best model by simulate the same amount of predicted data as observed in the experiment using posterior estimates of the model parameters. There was very good agreement between the observed data and the model predictions across conditions and sessions (Figure 5).

### Hierarchical drift-diffusion model analyses

**Figure 6:**
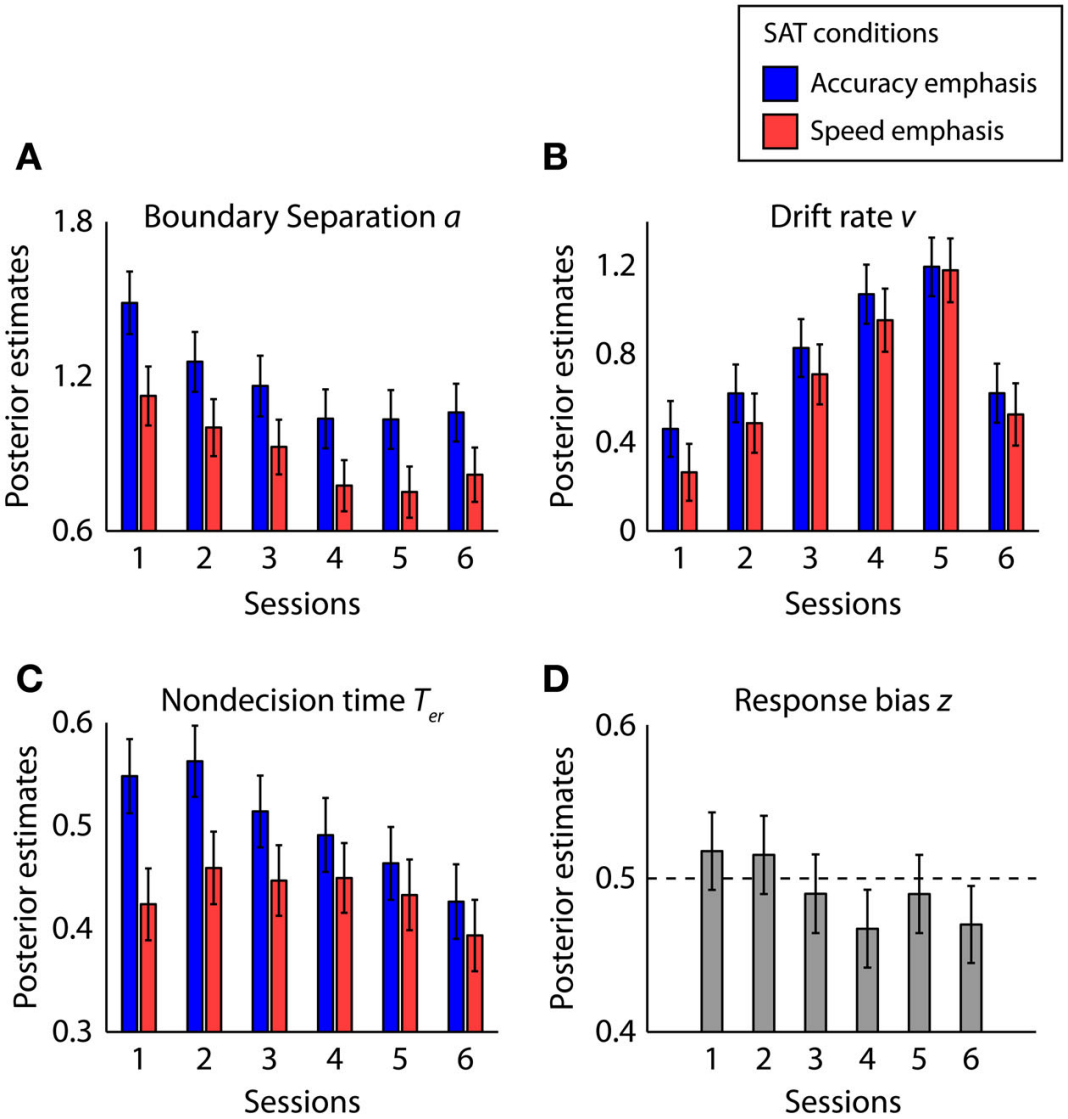
**Posterior estimates of the hierarchal drift-diffusion model parameters: (A) boundary separation *a*, (B) drift rate *v*, (C) non-decision time and *T*_*er*_ and (D) response bias *z***. The bars are the sampled mean posterior estimates and the error bars are standard deviations from sampled posterior distributions.

The hierarchical DDM incorporates parameters estimates (*a, v, T*_*er*_, and *z*) at the individual-subject level and population estimates of these parameters at the group level (Wiecki et al., 2013). We used two complementary approaches to determine the effects of speed-accuracy instructions and learning on the model parameters. First, for each parameter at the individual-subject level, the mean of its posterior distribution was used as a point estimate for group analysis. Second, for each group-level parameter, the mean and the standard deviation of its posterior distribution were used to quantify group-level measures and estimation uncertainties (Figure 6). We also used the group-level posteriors to compare two parameters in Bayesian methodology (Lindley, 1965; Berger and Bayarri, 2004; Kruschke, 2010, see section Data Processing and Analysis for details). For simplicity, below we used *p* to refer to classical frequentist *p*-value from ANOVA, and *P*_*P|D*_ to refer to the proportion of the posteriors supporting the testing hypothesis at the group level.

#### Boundary separation

Figure 6A showed the posterior mean and standard deviation of the boundary separation for each task condition and session. The boundary separation was significantly larger in the accuracy conditions than in the speed conditions [*F*_(1, 5)_ = 16.21, *p* < 0.01, partial η^2^ = 0.76, *P*_*P|D*_ = 0.95]. *Post-hoc* tests showed significant differences between SAT conditions in all sessions (*p* < 0.05, Wilcoxon signed ranks test, *P*_*P|D*_ > 0.93). The interaction between the SAT condition and session is not significant [*F*_(5, 25)_ = 0.34, *p* = 0.89 partial η^2^ = 0.06], suggesting similar extent of the speed-accuracy effect on boundary separation across sessions.

There is a main effect of session [*F*_(5, 25)_ = 7.83, *p* < 0.001, partial η^2^ = 0.61]. Learning at the trained directions gradually decreases boundary separation, as supported by a linear effect in the first five sessions [*F*_(1, 5)_ = 15.17, *p* < 0.05, partial η^2^ = 0.75]. Boundary separation at untrained directions after learning (session 6) is lower than that at the first session [*F*_(1, 5)_ = 9.41, *p* < 0.05, partial η^2^ = 0.65, *P*_*P|D*_ = 0.98], but similar to the parameter at the trained directions after learning (session 5) [*F*_(1, 5)_ = 1.68, *p* = 0.25, partial η^2^ = 0.25, *P*_*P|D*_ = 0.37]. Therefore, the learning effect on boundary separation generalized between trained and untrained directions.

#### Drift rate

The mean drift rate (Figure 6B) did not significantly differ between SAT conditions across all sessions [*F*_(1, 5)_ = 2.93, *p* = 0.15, partial η^2^ = 0.37, *P*_*P|D*_ = 0.76], consistent with our model comparison result that the mean drift rate is not the main factor in explaining the effects of speed-accuracy instructions. Interestingly, there was a marginal interaction effect between task conditions and sessions before and after training (sessions 1 and 5) [*F*_(5, 25)_ = 6.14, *p* = 0.06, partial η^2^ = 0.55], which is mainly driven by the higher mean drift rate in the accuracy condition than the speed condition in the first session (*p* < 0.05, Wilcoxon signed ranks test, *P*_*P|D*_ = 0.86).

The main effect of session on the mean drift rate was significant [*F*_(5, 25)_ = 118.50, *p* < 0.00001, partial η^2^ = 0.96], with a linear increase in the first five sessions at the trained directions [*F*_(1, 5)_ = 350.98, *p* < 0.00001, partial η^2^]. The drift rate at the untrained directions was lower than that at the trained directions after learning [*F*_(1, 5)_ = 217.53, *p* < 0.00001, partial η^2^ = 0.98, *P*_*P|D*_ ≈1], consistent with the observed data that improvements in accuracy did not transfer to the untrained directions after learning.

#### Non-decision time

The non-decision time (Figure 6C) was larger in the accurate condition than in the speed condition [*F*_(1, 5)_ = 8.21, *p* < 0.05, partial η^2^ = 0.62, *P*_*P|D*_ = 0.89]. Pairwise comparison within each session indicates that the effects of speed-accuracy instructions were significant in the first three sessions (*p* < 0.05, Wilcoxon signed ranks test, *P*_*P|D*_ > 0.91) but not in the last three sessions (*p* > 0.08, Wilcoxon signed ranks test, *P*_*P|D*_ < 0.80). No significant effect of session was observed [*F*_(5, 25)_ = 1.57, *p* = 0.21, partial η^2^ = 0.24], but there is an interaction between task conditions and sessions before and after training [*F*_(1, 5)_ = 6.83, *p* < 0.05, partial η^2^ = 0.58]. These results suggest that the speed-accuracy instructions affect the non-decision time at a larger extent at the beginning of training.

#### Response bias

The posterior estimates of the response bias were close to 0.5 in all sessions (Figure 6D) and a repeated-measures ANOVA showed no effect of sessions [*F*_(5, 25)_ = 0.78, *p* = 0.58, partial η^2^ = 0.13]. Therefore, there was no significant bias toward any of the two responses or change of biases across sessions.

## Discussion

This study examined how the two widely observed phenomenon, SAT and perceptual learning, differentially shape decision-making processes over different timescales and stages of learning. Speed emphasis or accuracy emphasis, in a coherent motion discrimination task, rapidly modulated participant's behavior between short blocks of trials (fast and error-prone or slow and accurate). This tradeoff between speed and accuracy was consistent throughout training and generalized between trained and untrained directions. The model analysis suggested that accuracy emphasis, compared with speed emphasis, not only increases the total amount of evidence required to render a decision (i.e., boundary separation), but also increases the quality of the evidence being accumulated (i.e., drift rate) and the latencies on stimulus encoding and motor preparation (i.e., non-decision time). Importantly, the effect of speed-accuracy instructions on boundary separation was significant across multiple sessions, but the effect on drift rate and non-decision time was significant only at the beginning of training.

One common assumption often made is that speed-accuracy instruction influences only the boundary separation. This selective influence assumption was largely accommodated by the ability of the constrained DDM with only varied boundaries to adequately fit behavioral data under SAT manipulations (Ratcliff and Rouder, 1998; Wagenmakers et al., 2008). However, such an approach cannot rule out possible influence of speed-accuracy instructions on other model parameters. Recent studies have considered more flexible models and identified the speed-accuracy effects on drift rate and non-decision time. By reanalyzing the data from Ratcliff and Rouder (1998), Vandekerckhove et al. (2011) suggested that the SAT is better described by changes in both drift rate and boundary separation than changes in boundary alone, with larger drift rate and boundary separation under accuracy emphasis. Similarly, Rae et al. (in press) reported that a constrained model with invariant drift rate between speed emphasis and accuracy emphasis conditions would underpredict the observed decision accuracy difference between the SAT conditions, which we also noticed from simulations of the inferior model (Model 3 in Figure 4). Rae et al. (in press) also reported larger drift rate change between speed-accuracy instructions in more difficult tasks than easier tasks. Interestingly, this is consistent with our result of significant drift rate change only in the first session, because the same task is relatively difficult for participants at the beginning of their training. Furthermore, studies using the DDM with variable non-decision time between different speed-accuracy conditions suggested decreased non-decision time when response speed is emphasized (Voss et al., 2004; Mulder et al., 2010, 2013). Therefore, emphasizing speed or accuracy affects multiple processes, not only the total amount of evidence needed for making a decision.

We found different effects of speed-accuracy instructions on the model parameters over the course of learning. For a difficult and unfamiliar task, emphasizing accuracy resulted in increased boundary separation, drift rate, and non-decision time. Once the participants learned the task after substantial training, the effect of speed-accuracy instructions was evident only on boundary separation. These findings confirmed a substantial role of boundary separation in response to speed-accuracy instructions (Ratcliff and Rouder, 1998; Wagenmakers et al., 2008; Starns and Ratcliff, 2014) throughout learning and generalized between trained and untrained stimulus features. The influence of speed-accuracy instructions on the other two DDM parameters is not intuitive, because unlike boundary separation, changing drift rate or non-decision time itself cannot describe an inverse relationship between decision error and RT as observed in SAT: increasing drift rate results in lower decision errors but shorter RT, and increasing non-decision time results in longer RT but no change in accuracy (Ratcliff and McKoon, 2008).

Nevertheless, several possible hypotheses may explain why learning influences the drift rate and non-decision time in response to speed-accuracy instructions. First, Rae et al. (in press) proposed that the quality of information extracted from the environment improves over the course of a single decision, and the rates of the changes are identical in both speed and accuracy emphasis conditions. Since the RT is smaller when response speed is emphasized, the drift rate estimated from the speed condition is largely based on the quality of information extracted early after stimulus onset, which would be systematically lower than the information quality later in a trial (i.e., as in the accuracy condition). Second, drift rate has been linked to the allocation of attention on the task (Schmiedek et al., 2007). It is possible that speed-accuracy instructions have impacts on the balance of attentional resources allocated between the decision process and other cognitive processes. For example, speed emphasis may facilitate the monitoring of elapsed time within a trial, which limits the attentional resources for extracting information for decision-making. Third, Rinkenauer et al. (2004) examined the SAT effects on lateralized readiness potentials (Leuthold et al., 1996; Eimer, 1998; Masaki et al., 2004) and observed decreased intervals between response-locked lateralized readiness potential onset and motor responses under speed emphasis (see Osman et al., 2000 for similar results). Since lateralized readiness potential intervals refer to the duration of motor processes after a decision being made, the findings from the electrophysiological data posit a role of speed-accuracy instructions on both decision and post-decision processes. This further supports our findings of decreased non-decision time under speed emphasis, because response execution is often considered an important component described by non-decision time in the DDM (Ratcliff and McKoon, 2008). However, it is not immediately clear why the SAT effects on drift rate and non-decision making are more evident at the beginning of training. An active account is that participants change their decision strategy after they become proficient about the procedure and the task (e.g., Adini et al., 2004). In other words, participants may learn to integrate information across larger periods of the stimulus presentation, decreasing the time spent on processes outside of decision-making and hence improving performance. Or, in a more passive account, because the task becomes much easier after training, there is only a limited capacity to improve on the accuracy and RT, which in turn limits the influence of speed-accuracy instructions on the model parameters other than boundary separation. Future investigations on how learning underpins the SAT at various task difficulty levels are necessary.

Our results demonstrated distinct perceptual learning mechanisms with different properties. As expected, training with feedback led to gradual improvements in decision accuracy and speed. The learning effect on accuracy was specific to the trained directions (Liu and Weinshall, 2000), but the improvement on RT partially generalized to untrained directions after training. Unlike most previous perceptual learning studies, which have focused only on decision accuracy but ignored decision speed (e.g., Fahle and Poggio, 2002; Dosher and Lu, 2007), we used the DDM to provide a mechanistic interpretation of both accuracy and speed improvements during learning (see Dutilh et al., 2009, 2011; Petrov et al., 2011; Liu and Watanabe, 2012 for similar approaches). Drift rate increased over training and the increase was specific to the trained directions, compatible with the theory that sensory processing is enhanced after learning (Karni and Sagi, 1991; Gilbert et al., 2001). This is also consistent with neurophysiological evidence that improved behavioral performance over training is accompanied by changes in sensory-driven responses of neurons in areas associated with perceptual decisions (Law and Gold, 2008). Boundary separation decreased over training and did not significantly differ between trained and untrained directions after training. Therefore, after substantial training of two motion directions, less accumulated evidence is required to discriminate coherent motion between two novel directions, even though the quality of extracted information from novel stimulus (e.g., drift rate for untrained directions) is lower. These findings further confirmed previous studies showing the learning effect on drift rate and boundary separation (Petrov et al., 2011; Liu and Watanabe, 2012).

The current study highlighted the benefits of using Bayesian methods to implement the DDM with the recently proposed hierarchical extension (Vandekerckhove et al., 2011; Wiecki et al., 2013). The hierarchical DDM is powerful in recovering model parameters with limited observed data (e.g., Jahfari et al., 2013). This feature is particularly important for the current study, because data from different training sessions need to be considered separately. One major advantage of using Bayesian methods for parameter estimation is the practicality of the obtained posterior parameter distributions. As we demonstrated in the current study, the posterior distributions can either be used to provide point estimates for classical frequentist inference, or can be directly used for Bayesian inference at both individual and group levels.

Two issues require further consideration. First, the drift-diffusion model is only an exemplar model of a large family of sequential sampling models (Ratcliff and Smith, 2004; Smith and Ratcliff, 2004; Bogacz et al., 2006; Zhang, 2012), and there are also simplified accumulator models omitting the noise in momentary evidence (Brown and Heathcote, 2005, 2008). These models mainly differ in how evidence supporting different alternatives is accumulated over time. It is of theoretical interest to explore whether our findings depend on the specific structure of the models we used. For example, one recent study showed similar influence of speed-accuracy instructions on model parameters in the DDM and in an accumulator model (Rae et al., in press). Second, we used a combination of bonuses and warning messages to help participants engage in the task, which is similar to early studies using a payoff matrix with criterion time (Fitts, 1966; Pachella and Pew, 1968) This design has been proven to be efficient in modulating behavior (Dutilh et al., 2009; Petrov et al., 2011). However, it is possible that participants would adopt a different decision strategy if the feedback or payoff is changed (e.g., the ratio of correct and error bonuses, see Simen et al., 2006, 2009; Bogacz et al., 2010; Balci et al., 2011).

In summary, we showed that the influence of speed-accuracy instructions cannot be attributed to a single change in decision boundary, but also relates to changes in other parameters that are relevant to the decision-making process and depends on the stage of learning. Future research on this topic should therefore take into account the complexity of individual's response to speed-accuracy instructions.

### Conflict of interest statement

The authors declare that the research was conducted in the absence of any commercial or financial relationships that could be construed as a potential conflict of interest.

## Footnotes

^1^Conventionally, a DIC difference of more than 10 indicates that the evidence supporting the best model is substantial (Burnham and Anderson, 2002). Because the difference of DIC values between the best and the second best model is close to this criterion, we repeated the same analysis on parameters estimates as in section hierarchical drift-diffusion model analyses for the second best model. The parameter changes between task conditions and sessions remain significant in the second best model.

## References

[B1] Adini Y. Wilkonsky A. Haspel R. Tsodyks M. Sagi D. (2004). Perceptual learning in contrast discrimination: the effect of contrast uncertainty. J. Vis. 4, 993–1005 10.1167/4.12.2 15669907

[B2] Ahissar M. Hochstein S. (1997). Task difficulty and the specificity of perceptual learning. Nature 387, 401–406 10.1038/387401a0 9163425

[B3] Ahissar M. Hochstein S. (2004). The reverse hierarchy theory of visual perceptual learning. Trends Cogn. Sci. 8, 457–464 10.1016/j.tics.2004.08.011 15450510

[B4] Balci F. Simen P. Niyogi R. Saxe A. Hughes J. A. Holmes P. (2011). Acquisition of decision making criteria: reward rate ultimately beats accuracy. Atten. Percept. Psychophys. 73, 640–657 10.3758/s13414-010-0049-7 21264716 PMC3383845

[B5] Bao M. Yang L. Rios C. He B. Engel S. A. (2010). Perceptual learning increases the strength of the earliest signals in visual cortex. J. Neurosci. 30, 15080–15084 10.1523/JNEUROSCI.5703-09.2010 21068313 PMC3073503

[B6] Barnett S. D. Heinemann A. W. Libin A. Houts A. C. Gassaway J. Sen-Gupta S. (2012). Small N designs for rehabilitation research. J. Rehabil. Res. Dev. 49, 175–186 10.1682/JRRD.2010.12.0242 22492346

[B7] Beersma B. Hollenbeck J. R. Humphrey S. E. Moon H. Conlon D. E. Ilgen D. R. (2003). Cooperation, competition, and team performance: toward a contingency approach. Acad. Manag. J. 46, 572–590 10.2307/30040650

[B8] Berger J. O. Bayarri M. J. (2004). The Interplay of Bayesian and frequentist analysis. Stat. Sci. 19, 58–80 10.1214/08834230400000011622578440

[B9] Blumen H. M. Gazes Y. Habeck C. Kumar A. Steffener J. Rakitin B. C. (2011). Neural networks associated with the speed-accuracy tradeoff: evidence from the response signal method. Behav. Brain Res. 224, 397–402 10.1016/j.bbr.2011.06.004 21699922 PMC3159733

[B10] Bogacz R. Brown E. Moehlis J. Holmes P. Cohen J. D. (2006). The physics of optimal decision making: a formal analysis of models of performance in two-alternative forced-choice tasks. Psychol. Rev. 113, 700–765 10.1037/0033-295X.113.4.700 17014301

[B11] Bogacz R. Hu P. T. Holmes P. J. Cohen J. D. (2010). Do humans produce the speed-accuracy trade-off that maximizes reward rate? Q. J. Exp. Psychol. 63, 863–891 10.1080/17470210903091643 19746300 PMC2908414

[B12] Brainard D. H. (1997). The psychophysics toolbox. Spat. Vis. 10, 433–436 10.1163/156856897X00357 9176952

[B13] Britten K. H. Shadlen M. N. Newsome W. T. Movshon J. A. (1993). Responses of neurons in macaque MT to stochastic motion signals. Vis. Neurosci. 10, 1157–1169 10.1017/S0952523800010269 8257671

[B15] Brown S. Heathcote A. (2005). A ballistic model of choice response time. Psychol. Rev. 112, 117–128 10.1037/0033-295X.112.1.117 15631590

[B14] Brown S. D. Heathcote A. (2008). The simplest complete model of choice response time: linear ballistic accumulation. Cogn. Psychol. 57, 153–178 10.1016/j.cogpsych.2007.12.002 18243170

[B16] Bulté I. Onghena P. (2008). An R package for single-case randomization tests. Behav. Res. Methods 40, 467–478 10.3758/BRM.40.2.467 18522057

[B17] Burnham K. Anderson D. (2002). Model Selection and Multimodel Inference: a Practical Information-Theoretic Approach, 2nd Edn. New York, NY: Springer-Verlag

[B18] Chittka L. Dyer A. G. Bock F. Dornhaus A. (2003). Psychophysics: bees trade off foraging speed for accuracy. Nature 424:388 10.1038/424388a 12879057

[B19] Chittka L. Skorupski P. Raine N. E. (2009). Speed-accuracy tradeoffs in animal decision making. Trends Ecol. Evol. 24, 400–407 10.1016/j.tree.2009.02.010 19409649

[B20] Coolican H. (2009). Research Methods and Statistics in Psychology, 5th Edn. London: Hodder Education

[B21] Dosher B. A. Lu Z.-L. (2007). The functional form of performance improvements in perceptual learning: learning rates and transfer. Psychol. Sci. 18, 531–539 10.1111/j.1467-9280.2007.01934.x 17576267

[B22] Dutilh G. Krypotos A.-M. Wagenmakers E.-J. (2011). Task-related versus stimulus-specific practice. Exp. Psychol. 58, 434–442 10.1027/1618-3169/a000111 21592942

[B23] Dutilh G. Vandekerckhove J. Tuerlinckx F. Wagenmakers E.-J. (2009). A diffusion model decomposition of the practice effect. Psychon. Bull. Rev. 16, 1026–1036 10.3758/16.6.1026 19966251

[B24] Edgington E. (1995). Randomization Tests, 4th Edn. New York, NY: Marcel Dekker

[B25] Eimer M. (1998). The lateralized readiness potential as an on-line measure of central response activation processes. Behav. Res. Methods Instrum. Comput. 30, 146–156 10.3758/BF03209424

[B26] Fahle M. (2005). Perceptual learning: specificity versus generalization. Curr. Opin. Neurobiol. 15, 154–160 10.1016/j.conb.2005.03.010 15831396

[B27] Fahle M. Poggio T. (2002). Perceptual Learning. Cambridge: MIT Press

[B28] Fitts P. M. (1966). Cognitive aspects of information processing: III. Set for speed versus accuracy. J. Exp. Psychol. 71, 849–857 10.1037/h0023232 5939364

[B29] Forstmann B. U. Dutilh G. Brown S. Neumann J. von Cramon D. Y. Ridderinkhof K. R. (2008). Striatum and pre-SMA facilitate decision-making under time pressure. Proc. Natl. Acad. Sci. U.S.A. 105, 17538–17542 10.1073/pnas.0805903105 18981414 PMC2582260

[B30] Franks N. R. Dornhaus A. Fitzsimmons J. P. Stevens M. (2003). Speed versus accuracy in collective decision making. Philos. Trans. R. Soc. B Biol. Sci. 270, 2457–2463 10.1098/rspb.2003.2527 14667335 PMC1691524

[B31] Furmanski C. S. Schluppeck D. Engel S. A. (2004). Learning strengthens the response of primary visual cortex to simple patterns. Curr. Biol. 14, 573–578 10.1016/j.cub.2004.03.032 15062097

[B32] Gamerman D. Lopes H. F. (2006). Markov Chain Monte Carlo: Stochastic Simulation for Bayesian Inference. 2nd Edn London: Taylor and Francis

[B33] Gelman A. Carlin J. Stern H. Rubin D. (2004). Bayesian Data Analysis, 2nd Edn. Boca Raton, FL: Chapman & Hall/CRC Press

[B34] Gelman A. Rubin D. B. (1992). Inference from iterative simulation using multiple sequences. Stat. Sci. 7, 457–472 10.1214/ss/1177011136

[B35] Gilbert C. D. Sigman M. Crist R. E. (2001). The neural basis of perceptual learning. Neuron 31, 681–697 10.1016/S0896-6273(01)00424-X 11567610

[B37] Gold J. I. Shadlen M. N. (2007). The neural basis of decision making. Annu. Rev. Neurosci. 30, 535–574 10.1146/annurev.neuro.29.051605.113038 17600525

[B38] Green C. S. Bavelier D. (2003). Action video game modifies visual selective attention. Nature 423, 534–537 10.1038/nature01647 12774121

[B39] Hanks T. D. Ditterich J. Shadlen M. N. (2006). Microstimulation of macaque area LIP affects decision-making in a motion discrimination task. Nat. Neurosci. 9, 682–689 10.1038/nn1683 16604069 PMC2770004

[B40] Heathcote A. Brown S. Mewhort D. J. K. (2000). The power law repealed: the case for an exponential law of practice. Psychon. Bull. Rev. 7, 185–207 10.3758/BF03212979 10909131

[B41] Heekeren H. R. Marrett S. Ungerleider L. G. (2008). The neural systems that mediate human perceptual decision making. Nat. Rev. Neurosci. 9, 467–479 10.1038/nrn2374 18464792

[B42] Heitz R. P. Schall J. D. (2012). Neural mechanisms of speed-accuracy tradeoff. Neuron 76, 616–628 10.1016/j.neuron.2012.08.030 23141072 PMC3576837

[B43] Ho T. C. Brown S. Serences J. T. (2009). Domain general mechanisms of perceptual decision making in human cortex. J. Neurosci. 29, 8675–8687 10.1523/JNEUROSCI.5984-08.2009 19587274 PMC2719543

[B44] Huk A. C. Shadlen M. N. (2005). Neural activity in macaque parietal cortex reflects temporal integration of visual motion signals during perceptual decision making. J. Neurosci. 25, 10420–10436 10.1523/JNEUROSCI.4684-04.2005 16280581 PMC6725829

[B45] Ivanoff J. Branning P. Marois R. (2008). fMRI evidence for a dual process account of the speed-accuracy tradeoff in decision-making. PLoS ONE 3:e2635 10.1371/journal.pone.0002635 18612380 PMC2440815

[B46] Jahfari S. Ridderinkhof K. R. Scholte H. S. (2013). Spatial frequency information modulates response inhibition and decision-making processes. PLoS ONE 8:e76467 10.1371/journal.pone.0076467 24204630 PMC3804599

[B87] Karni A. Sagi D. (1991). Where practice makes perfect in texture discrimination: evidence for primary visual cortex plasticity. Proc. Natl. Acad. Sci. U.S.A. 88, 4966–4970 10.1073/pnas.88.11.4966 2052578 PMC51788

[B47] Kayser A. S. Erickson D. T. Buchsbaum B. R. D'Esposito M. (2010). Neural representations of relevant and irrelevant features in perceptual decision making. J. Neurosci. 30, 15778–15789 10.1523/JNEUROSCI.3163-10.2010 21106817 PMC3020592

[B48] Kim J. N. Shadlen M. N. (1999). Neural correlates of a decision in the dorsolateral prefrontal cortex of the macaque. Nat. Neurosci. 2, 176–185 10.1038/5739 10195203

[B49] Kruschke J. (2010). Doing Bayesian Data Analysis: A Tutorial Introduction with R and BUGS. San Diego, CA: Academic Press/Elsevier

[B50] Laming D. R. J. (1968). Information Theory of Choice-Reaction Times. Oxford, UK: Academic Press

[B51] Law C.-T. Gold J. I. (2008). Neural correlates of perceptual learning in a sensory-motor, but not a sensory, cortical area. Nat. Neurosci. 11, 505–513 10.1038/nn2070 18327253 PMC2424192

[B52] Lehmann E. (1959). Testing Statistical Hypotheses. New York, NY: Wiley

[B53] Leuthold H. Sommer W. Ulrich R. (1996). Partial advance information and response preparation: inferences from the lateralized readiness potential. J. Exp. Psychol. Gen. 125, 307–323 10.1037/0096-3445.125.3.307 8830109

[B55] Lindley D. V. (1965). Introduction to Probability and Statistics from a Bayesian Viewpoint. Cambridge: Cambridge University Press 10.1017/CBO9780511662973

[B54] Li W. Piëch V. Gilbert C. D. (2004). Perceptual learning and top-down influences in primary visual cortex. Nat. Neurosci. 7, 651–657 10.1038/nn1255 15156149 PMC1440483

[B56] Link S. W. (1975). The relative judgment theory of two choice response time. J. Math. Psychol. 12, 114–135 10.1016/0022-2496(75)90053-X9305325

[B57] Link S. W. Heath R. A. (1975). A sequential theory of psychological discrimination. Psychometrika 40, 77–105 10.1007/BF02291481

[B58] Liu C. C. Watanabe T. (2012). Accounting for speed-accuracy tradeoff in perceptual learning. Vision Res. 61, 107–114 10.1016/j.visres.2011.09.007 21958757 PMC3288618

[B59] Liu Z. Weinshall D. (2000). Mechanisms of generalization in perceptual learning. Vision Res. 40, 97–109 10.1016/S0042-6989(99)00140-6 10768045

[B60] Logan G. D. (1992). Shapes of reaction-time distributions and shapes of learning curves: a test of the instance theory of automaticity. J. Exp. Psychol. Learn. Mem. Cogn. 18, 883–914 10.1037/0278-7393.18.5.883 1402715

[B61] Luce R. D. (1986). Response Times: Their Role in Inferring Elementary Mental Organization. New York, NY: Oxford University Press

[B62] Marshall J. A. R. Dornhaus A. Franks N. R. Kovacs T. (2006). Noise, cost and speed-accuracy trade-offs: decision-making in a decentralized system. J. R. Soc. Interface 3, 243–254 10.1098/rsif.2005.0075 16849234 PMC1578745

[B63] Masaki H. Wild-Wall N. Sangals J. Sommer W. (2004). The functional locus of the lateralized readiness potential. Psychophysiology 41, 220–230 10.1111/j.1469-8986.2004.00150.x 15032987

[B64] Matzke D. Dolan C. V. Logan G. D. Brown S. D. Wagenmakers E.-J. (2013). Bayesian parametric estimation of stop-signal reaction time distributions. J. Exp. Psychol. Gen. 142, 1047–1073 10.1037/a0030543 23163766

[B65] Mazurek M. E. Roitman J. D. Ditterich J. Shadlen M. N. (2003). A role for neural integrators in perceptual decision making. Cereb. Cortex 13, 1257–1269 10.1093/cercor/bhg097 14576217

[B66] Mulder M. J. Bos D. Weusten J. M. H. van Belle J. van Dijk S. C. Simen P. (2010). Basic impairments in regulating the speed-accuracy tradeoff predict symptoms of attention-deficit/hyperactivity disorder. Biol. Psychiatry 68, 1114–1119 10.1016/j.biopsych.2010.07.031 20926067

[B67] Mulder M. J. Keuken M. C. van Maanen L. Boekel W. Forstmann B. U. Wagenmakers E.-J. (2013). The speed and accuracy of perceptual decisions in a random-tone pitch task. Atten. Percept. Psychophys. 75, 1048–1058 10.3758/s13414-013-0447-8 23572205 PMC3691469

[B68] Nosofsky R. M. Palmeri T. J. (1997). An exemplar-based random walk model of speeded classification. Psychol. Rev. 104, 266–300 10.1037/0033-295X.104.2.266 9127583

[B69] Osman A. Lou L. Muller-Gethmann H. Rinkenauer G. Mattes S. Ulrich R. (2000). Mechanisms of speed–accuracy tradeoff: evidence from covert motor processes. Biol. Psychol. 51, 173–199 10.1016/S0301-0511(99)00045-9 10686365

[B70] Pachella R. G. Pew R. W. (1968). Speed-accuracy tradeoff in reaction time: effect of discrete criterion times. J. Exp. Psychol. 76, 19–24 10.1037/h0021275

[B71] Palmer J. Huk A. C. Shadlen M. N. (2005). The effect of stimulus strength on the speed and accuracy of a perceptual decision. J. Vis. 5, 376–404 10.1167/5.5.1 16097871

[B72] Petrov A. A. Van Horn N. M. Ratcliff R. (2011). Dissociable perceptual-learning mechanisms revealed by diffusion-model analysis. Psychon. Bull. Rev. 18, 490–497 10.3758/s13423-011-0079-8 21394547

[B73] Pilly P. K. Seitz A. R. (2009). What a difference a parameter makes: a psychophysical comparison of random dot motion algorithms. Vision Res. 49, 1599–1612 10.1016/j.visres.2009.03.019 19336240 PMC2789308

[B74] Ploran E. J. Nelson S. M. Velanova K. Donaldson D. I. Petersen S. E. Wheeler M. E. (2007). Evidence accumulation and the moment of recognition: dissociating perceptual recognition processes using fMRI. J. Neurosci. 27, 11912–11924 10.1523/JNEUROSCI.3522-07.2007 17978031 PMC6673371

[B75] Rae B. Heathcote A. Donkin C. Averell L. Brown S. (in press). The hare and the tortoise: emphasizing speed can change the evidence used to make decisions. J. Exp. Psychol. Learn. Mem. Cogn. 10.1037/a0036801 24797438

[B76] Ratcliff R. (1978). A theory of memory retrieval. Psychol. Rev. 85, 59–108 10.1037/0033-295X.85.2.59

[B77] Ratcliff R. (2002). A diffusion model account of response time and accuracy in a brightness discrimination task: fitting real data and failing to fit fake but plausible data. Psychon. Bull. Rev. 9, 278–291 10.3758/BF03196283 12120790

[B78] Ratcliff R. Gomez P. McKoon G. (2004). A diffusion model account of the lexical decision task. Psychol. Rev. 111, 159–182 10.1037/0033-295X.111.1.159 14756592 PMC1403837

[B79] Ratcliff R. McKoon G. (2008). The diffusion decision model: theory and data for two-choice decision tasks. Neural Comput. 20, 873–922 10.1162/neco.2008.12-06-420 18085991 PMC2474742

[B80] Ratcliff R. Rouder J. N. (1998). Modeling response times for two-choice decisions. Psychol. Sci. 9, 347–356 10.1111/1467-9280.00067

[B81] Ratcliff R. Rouder J. N. (2000). A diffusion model account of masking in two-choice letter identification. J. Exp. Psychol. Hum. Percept. Perform. 26, 127–140 10.1037/0096-1523.26.1.127 10696609

[B82] Ratcliff R. Smith P. L. (2004). A comparison of sequential sampling models for two-choice reaction time. Psychol. Rev. 111, 333–367 10.1037/0033-295X.111.2.333 15065913 PMC1440925

[B83] Ratcliff R. Tuerlinckx F. (2002). Estimating parameters of the diffusion model: approaches to dealing with contaminant reaction times and parameter variability. Psychon. Bull. Rev. 9, 438–481 10.3758/BF03196302 12412886 PMC2474747

[B84] Ratcliff R. Van Zandt T. McKoon G. (1999). Connectionist and diffusion models of reaction time. Psychol. Rev. 106, 261–300 10.1037/0033-295X.106.2.261 10378014

[B85] Rinkenauer G. Osman A. Ulrich R. Muller-Gethmann H. Mattes S. (2004). On the locus of speed-accuracy trade-off in reaction time: inferences from the lateralized readiness potential. J. Exp. Psychol. Gen. 133, 261–282 10.1037/0096-3445.133.2.261 15149253

[B86] Roitman J. D. Shadlen M. N. (2002). Response of neurons in the lateral intraparietal area during a combined visual discrimination reaction time task. J. Neurosci. 22, 9475–9489 12417672 10.1523/JNEUROSCI.22-21-09475.2002 PMC6758024

[B88] Schall J. D. (2002). The neural selection and control of saccades by the frontal eye field. Philos. Trans. R. Soc. B Biol. Sci. 357, 1073–1082 10.1098/rstb.2002.1098 12217175 PMC1693021

[B89] Schmiedek F. Oberauer K. Wilhelm O. Süss H.-M. Wittmann W. W. (2007). Individual differences in components of reaction time distributions and their relations to working memory and intelligence. J. Exp. Psychol. Gen. 136, 414–429 10.1037/0096-3445.136.3.414 17696691

[B90] Schouten J. F. Bekker J. A. M. (1967). Reaction time and accuracy. Acta Psychol. (Amst.) 27, 143–153 10.1016/0001-6918(67)90054-6 6062205

[B91] Shadlen M. N. Newsome W. T. (2001). Neural basis of a perceptual decision in the parietal cortex (Area LIP) of the rhesus monkey. J. Neurophysiol. 86, 1916–1936 11600651 10.1152/jn.2001.86.4.1916

[B92] Simen P. Cohen J. D. Holmes P. (2006). Rapid decision threshold modulation by reward rate in a neural network. Neural Netw. 19, 1013–1026 10.1016/j.neunet.2006.05.038 16987636 PMC1808344

[B93] Simen P. Contreras D. Buck C. Hu P. Holmes P. Cohen J. D. (2009). Reward rate optimization in two-alternative decision making: empirical tests of theoretical predictions. J. Exp. Psychol. Hum. Percept. Perform. 35, 1865–1897 10.1037/a0016926 19968441 PMC2791916

[B94] Smith P. L. Ratcliff R. (2004). Psychology and neurobiology of simple decisions. Trends Neurosci. 27, 161–168 10.1016/j.tins.2004.01.006 15036882

[B95] Spiegelhalter D. J. Best N. G. Carlin B. P. van der Linde A. (2002). Bayesian measures of model complexity and fit. J. R. Stat. Soc. B Stat. Methodol. 64, 583–639 10.1111/1467-9868.00353

[B96] Starns J. J. Ratcliff R. (2014). Validating the unequal-variance assumption in recognition memory using response time distributions instead of ROC functions: a diffusion model analysis. J. Mem. Lang. 70, 36–52 10.1016/j.jml.2013.09.005 24459327 PMC3896247

[B97] Stone M. (1960). Models for choice-reaction time. Psychometrika 25, 251–260 10.1007/BF02289729

[B98] Townsend J. T. Ashby F. (1983). The Stochastic Modeling of Elementary Psychological Processes. Cambridge, MA: Cambridge University Press

[B99] Trobalon J. B. Chamizo V. D. Mackintosh N. J. (1992). Role of context in perceptual learning in maze discriminations. Q. J. Exp. Psychol. B. 44, 57–73 1546184 10.1080/02724999208250602

[B100] Uchida N. Mainen Z. F. (2003). Speed and accuracy of olfactory discrimination in the rat. Nat. Neurosci. 6, 1224–1229 10.1038/nn1142 14566341

[B101] Vandekerckhove J. Tuerlinckx F. Lee M. D. (2011). Hierarchical diffusion models for two-choice response times. Psychol. Methods 16, 44–62 10.1037/a0021765 21299302

[B102] Van Veen V. Krug M. K. Carter C. S. (2008). The neural and computational basis of controlled speed-accuracy tradeoff during task performance. J. Cogn. Neurosci. 20, 1952–1965 10.1162/jocn.2008.20146 18416686

[B103] Voss A. Rothermund K. Voss J. (2004). Interpreting the parameters of the diffusion model: an empirical validation. Mem. Cognit. 32, 1206–1220 10.3758/BF03196893 15813501

[B104] Wagenmakers E.-J. (2009). Methodological and empirical developments for the Ratcliff diffusion model of response times and accuracy. Eur. J. Cogn. Psychol. 21, 641–671 10.1080/09541440802205067

[B105] Wagenmakers E.-J. Ratcliff R. Gomez P. McKoon G. (2008). A diffusion model account of criterion shifts in the lexical decision task. J. Mem. Lang. 58, 140–159 10.1016/j.jml.2007.04.006 19122740 PMC2330283

[B106] Wald A. (1947). Sequential Analysis. New York, NY: Wiley

[B107] Watanabe T. Náñez J. E. Sasaki Y. (2001). Perceptual learning without perception. Nature 413, 844–848 10.1038/35101601 11677607

[B108] Wickelgren W. A. (1977). Speed-accuracy tradeoff and information processing dynamics. Acta Psychol. (Amst.) 41, 67–85 10.1016/0001-6918(77)90012-9

[B109] Wiecki T. V Sofer I. Frank M. J. (2013). HDDM: hierarchical bayesian estimation of the drift-diffusion model in python. Front. Neuroinform. 7:14 10.3389/fninf.2013.00014 23935581 PMC3731670

[B110] Wylie S. A. van den Wildenberg W. P. M. Ridderinkhof K. R. Bashore T. R. Powell V. D. Manning C. A. (2009). The effect of speed-accuracy strategy on response interference control in Parkinson's disease. Neuropsychologia 47, 1844–1853 10.1016/j.neuropsychologia.2009.02.025 19428416 PMC4524649

[B111] Yang T. Maunsell J. H. R. (2004). The effect of perceptual learning on neuronal responses in monkey visual area V4. J. Neurosci. 24, 1617–1626 10.1523/JNEUROSCI.4442-03.2004 14973244 PMC6730469

[B112] Zhang J. (2012). The effects of evidence bounds on decision-making: theoretical and empirical developments. Front. Psychol. 3:263 10.3389/fpsyg.2012.00263 22870070 PMC3409448

[B113] Zhang J. Hughes L. E. Rowe J. B. (2012). Selection and inhibition mechanisms for human voluntary action decisions. Neuroimage 63, 392–402 10.1016/j.neuroimage.2012.06.058 22776456 PMC3445813

[B114] Zhang J. Kourtzi Z. (2010). Learning-dependent plasticity with and without training in the human brain. Proc. Natl. Acad. Sci. U.S.A. 107, 13503–13508 10.1073/pnas.1002506107 20628009 PMC2922179

[B115] Zhang J. Meeson A. Welchman A. E. Kourtzi Z. (2010). Learning alters the tuning of functional magnetic resonance imaging patterns for visual forms. J. Neurosci. 30, 14127–14133 10.1523/JNEUROSCI.2204-10.2010 20962233 PMC6634776

